# Characteristics and Longitudinal Patterns of Erectile Dysfunction Drug Use Among Men Who Have Sex with Men in the U.S.

**DOI:** 10.1007/s10508-021-02065-x

**Published:** 2021-09-29

**Authors:** Jee Won Park, Adrian S. Dobs, Ken S. Ho, Frank J. Palella, Eric C. Seaberg, Robert E. Weiss, Roger Detels

**Affiliations:** 1 Department of Epidemiology, 71-269 CHS, Fielding School of Public Health, University of California, Los Angeles, CA 90095-1772 USA; 2 Department of Medicine, Johns Hopkins University School of Medicine, Baltimore, MD USA; 3 Department of Medicine, University of Pittsburg, Pittsburgh, PA USA; 4 Division of Infectious Diseases, Feinberg School of Medicine of Northwestern University, Chicago, IL USA; 5 Department of Epidemiology, Johns Hopkins Bloomberg School of Public Health, Baltimore, MD USA; 6 Department of Biostatistics, Fielding School of Public Health, University of California, Los Angeles, CA USA

**Keywords:** HIV, Phosphodiesterase 5 inhibitors, Recreational drugs, Sexual behavior, Multivariate analysis, Sexual orientation

## Abstract

We investigated the longitudinal relationship between erectile dysfunction (ED) drug use with behavioral factors, including substance use and sexual activities in men who have sex with men from the Multicenter AIDS Cohort Study during 1998–2016 (*n* = 1636). We used a bivariate random-intercept model to evaluate ED drug use along with other behavioral factors to assess relationships between the two outcomes over time on a population level and also at the individual level. Average ED drug use among men who have sex with men (MSM) with HIV was positively correlated with average use of marijuana (*r* = .19), poppers (*r* = .27), and stimulants (*r* = .25). In this group, testosterone use (*r* = .32), multiple partners (*r* = .41), insertive anal intercourse with condom (*r* = .40), and insertive anal intercourse without condom (*r* = .43) all showed moderate correlations over time with average ED use (*p* < .001). Associations among MSM without HIV were similar, with average marijuana use (*r* = .19) and stimulant use (*r* = .22) being positively correlated with average ED drug use, and were also correlated with having multiple partners (*r* = .36), insertive anal intercourse with condom (*r* = .22), and insertive anal intercourse without condom (*r* = .18) over time. Positive within-individual associations between ED drug use and multiple partners and insertive anal intercourse with and without condom were observed regardless of HIV serostatus. This study showed that MSM who reported use of ED drugs were also, on average, more likely to use recreational drugs and engage in sexual activities, such as having multiple partners and insertive anal intercourse. Within individuals, average ED drug use was also positively correlated with sexual behaviors.

## Introduction

Erectile dysfunction (ED) is common among men with HIV (MWH) (Romero-Velez et al., 2014) and is associated with positive HIV serostatus among men who have sex with men (MSM) (Dijkstra et al., 2018). Since initial approval by the United States (U.S.) Food and Drug Administration (FDA) in 1998, the use of ED drugs has been documented to be more common among MSM than among heterosexual men (Kim et al., 2002).

ED drugs are used by young men, as well as young MSM, as recreational drugs with simultaneous use of ED drugs and other recreational substances (Bechara et al., 2010; Fisher et al., 2006; Mansergh et al., 2006). However, the concurrent use of ED drugs with nitrate drugs or substances is unsafe as it may result in deleterious health effects, such as life-threatening hypotension (James, 1998; Lim et al., 2002; Purcell et al., 2005a, b). Despite the precautions and information, the use of recreational drugs is common among MSM regardless of age and socioeconomic status (Colfax et al., 2005; Mansergh et al., 2006; Purcell et al., 2005a, b), and these drugs include marijuana, cocaine, ecstasy, and ED drugs (Halkitis et al., 2005; Purcell et al., 2005a, b; Ruf et al., 2006). The majority of MSM from an Australian community cohort study reported that use of recreational drugs was part of their lifestyle (Ruf et al., 2006). In another cross-sectional study of young heterosexual, bisexual, and gay men in the U.S., although 5% of the participants reported using ED drugs, the majority were for recreational purposes (Harte & Meston, 2011). In the same study, slightly less than half of the men who had reported using ED drugs had combined the drugs together with marijuana and other substances such as ecstasy, methamphetamines, and cocaine.

ED drug use is closely related to engagement in sexual activities such as multiple sex partners and anal intercourse in MSM. Association between ED drug use and sexual behaviors has been reported in studies examining recreational drug use. Further, in a community-based sample of MSM, there was a strong association between ED drug use with use of illicit drugs and anal intercourse (Chu et al., 2003). Insertive anal intercourse was a major predictor of ED drug use, knowledge about one’s HIV serostatus, and numbers of sex partners among heterosexual men (Fisher et al., 2006). In other studies, insertive anal intercourse was associated with ED drug use (Fisher et al., 2011; Purcell et al., 2005a, b) (Spindler et al., 2007).

Thus, the higher likelihood of engaging in anal intercourse, having multiple partners, as well as the concurrent use of ED drugs and recreational drugs may increase the risk of HIV transmission (Schwarcz et al., 2007; Swearingen & Klausner, 2005). In general, to help reduce the transmission, studies and interventions that have examined ED drug use have focused on single targets, however, there is growing evidence that intervening on a single target may not be sufficient to reduce HIV transmission (Chu et al., 2003; Schwarcz et al., 2007; Swearingen & Klausner, 2005). While the relationships between ED drug use and other behaviors, such as recreational drug use and sexual behaviors, have been well described, the patterns of use over time among MSM are relatively unknown. Most studies to date regarding ED drug use and its associated factors among MSM and MWH have been cross-sectional.

Therefore, the purpose of this study was to assess the longitudinal patterns of ED drug use and their associations with other recreational drugs use, medications, and sexual behavior. We also examined associations between ED drug use and these other variables within individuals. Based on these studies, we hypothesized that ED drug use would be positively correlated over time with recreational drugs use and sexual activities, such as having multiple partners and insertive anal intercourse.

## Method

### Participants

We examined data collected from participants in the Multicenter AIDS Cohort Study (MACS) during semiannual interviews and physical examinations. MACS is an ongoing cohort study of MSM across 4 sites in the U.S. (Baltimore, Chicago, Pittsburgh, and Los Angeles) since 1984 (Detels et al., 1992; Kaslow et al., 1987). To examine the patterns of ED drug use in MWH and men without HIV (MWOH), we set our study initiation year to 1998, which was the year when the first ED drugs were available in the U.S. Thus, for the purposes of our study, we examined data from a total of 1,636 MSM who had been enrolled in the MACS study since 1998. Baseline for the MWH group was defined as the first visit by seropositive participants, or the first visit after seroconversion by HIV-seroconverted participants, at the start of, or after, the study initiation year (1998). Baseline for the MWOH group was the first visit by the seronegative participant at the start of, or after, the study initiation year.

### Measures

The main outcome variable of interest was self-reported use of ED drugs by participants since their last visit. Prescription drugs for ED treatment, such as sildenafil, tadalafil, and vardenafil were included as ED drugs, as well as other ED drugs that were not prescribed for ED treatment. This was a time-varying binary variable (yes/no).

Time-varying substance use since the last visit included self-reported use of marijuana, poppers (alkyl nitrites), stimulants (crack/cocaine, ecstasy, methamphetamine and uppers), heroin or other opiates, speedball (heroin and cocaine together), downers, ethyl chlorides (inhalants), GHB (gamma-hydroxybutyric acid), injection drugs, and testosterone. For all substance use variables, all missing values were replaced with a “no” if the participants during their next visit reported never using the drug.

Medication variables included information regarding the use of antiretroviral therapy (ART) at the time of visits (yes/no), self-reported depression medication use since the prior visit, and diabetes medications used since the prior visit.

Sexual behavior variables included the number of male and female partners since last visit and the number of partners since the last visit with whom the participants engaged in insertive anal intercourse with condom and insertive anal intercourse without condom. We transformed this information to create three binary variables describing sexual behavior: multiple partners (2 or more partners vs. 0 or 1), anal intercourse with condom (1 or more partners vs. 0), and anal intercourse without condom (1 or more partners vs. 0). All sexual behavior variables were self-reported.

Substance use, medication use, and sexual behaviors were treated as simultaneous outcomes, with ED drug use as the main outcome variable.

#### Demographic and Behavioral Characteristics

Covariates that were associated with the use of ED drugs were included as predictors in the longitudinal analysis. Demographic characteristics including age, race/ethnicity (non-Hispanic white, non-Hispanic black, and other), and educational attainment (college degree, and high school or less) were included in all analyses. Smoking status was a categorical variable divided into current, former and never smokers. Alcohol consumption was categorized into four groups: binge drinker (5 or more drinks per occasion at least once per month), moderate/heavy (more than 14 drinks per week), low/moderate (1–14 drinks per week), and none (0 drinks). All variables were self-reported, except for age, and time-varying, except race/ethnicity. BMI was a time-varying continuous measure that was categorized into three groups according to CDC’s adult BMI categories (Center for Disease Control & Prevention, 2017): normal (BMI < 25.0), overweight (25.0 ≤ BMI < 30.0), and obese (BMI ≥ 30.0).

HCV infection status was dichotomized into positive or negative, depending on the presence of serum HCV antibodies or plasma HCV RNA. Kidney disease was defined as having a current or prior confirmed diagnosis versus no diagnosis. Time-varying co-morbidities including pre-existing conditions included stroke, congestive heart failure, prostate surgery or cancer, or bladder surgery or cancer. These conditions were identified using ICD-9 codes, and were selected since they are known risk factors for ED and comprise contraindications for the use of ED drugs (Ferri et al., 2002; Lim et al., 2002; Miranda-Sousa et al., 2006). We then dichotomized the co-morbidity variable and defined it as the presence of at least one of the listed conditions or none.

### Statistical Analysis

*t*-test and chi-squared tests were used to examine bivariate associations between ED drug use at baseline and baseline demographic characteristics, substance use, health-related variables, and sexual behaviors.

We used bivariate generalized linear mixed models (GLMMs) to examine the association between pairs of response variables (Weiss, 2005), one of which was ED drug use, and the second was either recreational drug use, sexual behaviors, or medication use, each pair considered separately. This model has a parameter that describes the correlation between person-average levels of the two variables. All models were adjusted for age, race/ethnicity, education, smoking, alcohol consumption, BMI, kidney disease, HCV infection, and pre-existing conditions for both variables. These covariates were selected, a priori*,* based on past research on predictors of ED drug use and risk factors for ED (Chu et al., 2003; Ferri et al., 2002; Lim et al., 2002; Miranda-Sousa et al., 2006; Purcell et al., 2005a, b). Output from the model includes the variances and the covariances between the random intercepts, from which we calculated the correlation. P-values of the correlation are the same as that for the covariance.

To examine the association between ED drug use and response variables at particular time points within individuals, bivariate GLMMs of ED drug use and the variables were fit to obtain subject-specific time-varying residuals. The residuals were then included as predictors in the bivariate random-intercept model and the resulting sign, positive or negative, of the coefficient of the residual identified positive or negative within-subject time-varying associations.

All statistical analyses were performed with SAS 9.4 (SAS Institute Inc., Cary, NC).

## Results

From a total of 1,636 participants in this study, there were 62 men who seroconverted during the period of our study and contributed data to both the MWH and MWOH groups. Data from 1,391 MWH with 29,343 person-visits and 307 MWOH with 6,752 person-visits were included in the analysis. The median number of days between visits was 182 days for both MWH (interquartile range (IQR): 174–203) and MWOH groups (175–196). Median number of visits among MWH was 24 (IQR: 11–29) and 25 (15–28) among MWOH (Table 1).

**Table 1:** Median number of visits and days between each visit by MACS participants during 1998–2016

	Number of visits				Average days between visits				
	N	Median	*Q*1	*Q*3	Mean	SD	Median	*Q*1	*Q*3
MWH	1391	24	11	29	203.6	166.2	182	174	203
MWOH	307	25	15	28	191.8	107.2	182	175	196

*MWH* men with HIV, *MWOH* men without HIV, *N* number, *SD* standard deviation, *Q1* 25th percentile, *Q3* 75th percentile

Participants who seroconverted were also included in the MWH group (*n* = 62)

Table 2 shows descriptive statistics for ED drug users and non-users among MWH at baseline. Their mean age was 42.1 years (SD: 8.0). ED drug users were older than non-users. The majority of the participants were white (56.6%), had less than a four-year college degree (54.1%), and a normal BMI (54.9%). Marijuana use was similar in both ED drug users and non-users (39.0% vs. 41.3%), but a higher proportion of men who reported ED drug use also reported using poppers (41.5% vs. 26.1%) and stimulants (38.6% vs. 23.0%). Half of the subjects were on ART at baseline, with no significant differences between ED drug users and non-users. One in ten subjects had HCV infection at baseline, and this was more common among ED drug users (*p* = .003). The majority of participants at baseline reported having more than 2 sex partners since the last visit (53.0%), and a significantly higher proportion of ED drug users than ED drug non-users reported having more than 2 sex partners (80.3% vs. 51.3%), anal intercourse with condom (67.5% vs. 41.8%) and anal intercourse without condom (41.0% vs. 17.4%) since their last visit (all *p* < .001). Similar characteristics were observed among MWOH subjects (Table 3). There were no significant differences between ED drug users and non-users regarding substance use. A higher proportion of men who used ED drugs reported anal intercourse with condom (65.6% vs 46.3%; *p* = .04) and anal intercourse without condom since last visit (35.5% vs. 27.8%; *p* = .37).

**Table 2:** Baseline demographic characteristics of MWH participants by ED drug use since prior visit

Variable	ED Drug Use		Total
	No *N* = 1,307 (94.0%)	Yes *N* = 84 (6.0%)	*N* = 1391 (100%)
Age** (mean ± SD)	41.9 ± 7.8	44.6 ± 9.6	42.1 ± 8.0
Race			
@White	740 (56.6)	47 (56.0)	787 (56.6)
@Black	358 (27.4)	26 (31.0)	384 (27.6)
@Other	209 (16.0)	11 (13.1)	220 (15.8)
Education (college or higher)	528 (46.0)	38 (45.2)	566 (45.9)
Smoking status			
@Current	508 (39.3)	40 (48.8)	548 (39.9)
@Former	417 (32.3)	21 (25.6)	438 (31.9)
@Never	367 (28.4)	21 (25.6)	388 (28.2)
Alcohol consumption			
@Binge	150 (11.7)	12 (14.6)	162 (11.8)
@Moderate/Heavy	287 (22.3)	21 (25.6)	308 (22.5)
@Low/Moderate	596 (46.3)	42 (51.2)	638 (46.6)
@None	255 (19.8)	7 (8.5)	262 (19.1)
BMI* (kg/m^2^)			
@Obese (≥ 30)	136 (10.8)	2 (2.4)	138 (10.3)
@Overweight (25–29.9)	431 (34.2)	36 (43.4)	467 (34.8)
@Normal (≤ 24.9)	692 (55.0)	45 (54.2)	737 (54.9)
Study site*			
@Baltimore	305 (23.3)	30 (35.7)	335 (24.1)
@Chicago	307 (23.5)	11 (13.1)	318 (22.9)
@Pittsburgh	256 (19.6)	19 (22.6)	275 (19.8)
@Los Angeles	439 (33.6)	24 (28.6)	463 (33.3)
Drug use			
@Marijuana	529 (41.3)	3232 (39.0)	561 (41.2)
@Poppers***	335 (26.1)	34 (41.5)	369 (27.1)
@Stimulants^a,^***	296 (23.0)	32 (38.6)	328 (23.9)
@Heroin/opiates	40 (3.1)	2 (2.5)	42 (3.1)
@Speedball	18 (1.4)	2 (2.4)	20 (1.5)
@Ethyl chloride	4 (0.3)	1 (1.3)	5 (0.4)
@GHB***	9 (0.7)	6 (7.5)	15 (1.1)
@Injection drugs use***	90 (7.1)	16 (19.5)	106 (7.8)
Diabetes medication	34 (2.6)	1 (1.2)	35 (2.5)
Depression medication	326 (24.9)	27 (32.1)	353 (25.4)
Kidney disease	4 (0.3)	1 (1.2)	5 (0.4)
Testosterone**	24 (1.8)	7 (8.3)	31 (2.2)
ART	695 (53.2)	36 (42.9)	731 (52.6)
HCV infection**	129 (9.9)	17 (20.2)	146 (10.5)
Pre-existing conditions^b^	70 (5.4)	5 (6.0)	75 (5.4)
Multiple partners*** (≥ 2)	656 (51.3)	65 (80.3)	721 (53.0)
Insertive anal intercourse with condom*** (≥ 1 partner)	529 (41.8)	54 (67.5)	583 (43.3)
Insertive anal intercourse without condom*** (≥ 1 partner)	220 (17.4)	32 (41.0)	252 (18.8)

All results are in *N* (%) unless otherwise stated

*MWH* men with HIV, *N* number, *SD* standard deviation, *GHB* gamma-hydroxybutyric acid, *ART* antiretroviral therapy, *HCV* hepatitis C virus

^a^Cocaine, ecstasy, methamphetamine, uppers

^b^Stroke, coronary heart failure, prostate surgery/cancer, bladder surgery/cancer

*p < .05. **p < .01. ***p < .001. *p* values were computed using *t*-test and chi-squared tests for bivariate association between ED drug use and the covariates

**Table 3:** Baseline demographic characteristics of MWOH participants by ED drug use since prior visit

Variable	ED drug use		Total
	No *n* = 274 (89.3%)	Yes *n* = 33 (10.7%)	*N* = 307 (100.0%)
Age* (mean ± SD)	43.9 ± 9.6	47.8 ± 9.7	44.4 ± 9.6
Race			
@White	148 (54.0)	19 (57.6)	167 (54.4)
@Black	100 (36.5)	9 (27.3)	109 (35.5)
@Other	26 (9.5)	5 (15.2)	31 (10.1)
Education (college or higher)	139 (51.1)	12 (36.4)	151 (49.5)
Smoking status			
@Current	119 (43.6)	10 (30.3)	129 (42.2)
@Former	79 (28.9)	16 (48.5)	95 (31.1)
@Never	75 (27.5)	7 (21.2)	82 (26.8)
Alcohol consumption			
@Binge	39 (14.3)	3 (9.1)	42 (13.7)
@Moderate/Heavy	69 (25.3)	6 (18.2)	75 (24.5)
@Low/Moderate	119 (43.6)	21 (63.6)	140 (45.8)
@None	46 (16.9)	3 (9.1)	49 (16.0)
BMI (kg/m^2^)			
@Obese (≥ 30)	54 (19.7)	3 (9.1)	57 (18.6)
@Overweight (25–29.9)	98 (35.8)	12 (36.4)	110 (35.8)
@Normal (≤ 24.9)	122 (44.5)	18 (54.6)	140 (45.6)
Study site			
@Baltimore	61 (22.3)	12 (36.4)	73 (23.8)
@Chicago	62 (22.6)	8 (24.2)	70 (22.8)
@Pittsburgh	89 (32.5)	9 (27.3)	98 (31.9)
@Los Angeles	62 (22.6)	4 (12.1)	66 (21.5)
Drug Use			
@Marijuana	98 (35.9)	15 (45.5)	113 (36.9)
@Poppers	59 (21.6)	12 (36.4)	71 (23.2)
@Stimulants^a,^*	65 (23.8)	13 (39.4)	78 (25.5)
@Heroin/opiates	21 (7.7)	3 (9.1)	24 (7.9)
@Speedball	13 (4.8)	1 (3.0)	14 (4.6)
@Ethyl chloride	1 (0.4)	0 (0.0)	1 (0.3)
@GHB**	1 (0.4)	1 (3.0)	2 (0.7)
@Injection drugs use	44 (16.2)	6 (18.2)	50 (16.4)
@Diabetes medication	7 (2.6)	3 (9.1)	10 (3.3)
@Depression medication	49 (17.9)	8 (24.2)	57 (18.6)
Kidney disease			
Testosterone			
ART			
HCV infection	55 (20.1)	7 (21.2)	62 (20.2)
Pre-existing conditions^b^	14 (5.1)	3 (9.1)	17 (5.5)
Multiple partners (≥ 2)	173 (63.4)	26 (78.8)	199 (65.0)
Insertive anal intercourse with condom* (≥ 1 partner)	125 (46.3)	21 (65.6)	146 (48.3)
Insertive anal intercourse without condom (≥ 1 partner)	75 (27.8)	11 (35.5)	86 (28.6)

All results are in N (%) unless otherwise stated

*MWOH* men without HIV, *N* number, *SD* standard deviation, *GHB* gamma-hydroxybutyric acid, *ART* antiretroviral therapy, *HCV* hepatitis C virus

^a^Cocaine, ecstasy, methamphetamine, uppers

^b^Stroke, coronary heart failure, prostate surgery/cancer, bladder surgery/cancer

*p < .05. **p < .01. ***p < .001. *p* values were computed using *t*-test and chi-squared tests for bivariate association between ED drug use and the covariates

Table 4 shows the results from bivariate random-intercept models with ED drug use and a second outcome variable among MWH. Average ED drug use was significantly correlated with average use of marijuana (*r* = .19), poppers (*r* = .27), and stimulants (*r* = .25), all with *p* < .001. GHB and heroin/opiate use did not converge due to small numbers of users, and are not reported in the table. Depression medication use also showed a significant though weak positive correlation with ED drug use (*r* = .08, *p* = .02). Testosterone use showed moderate correlations with ED drug use over time (*r* = .32, *p* < .001), and moderate significant correlations were found between ED drug use and the sexual behavior variables, including multiple partners (*r* = .41, *p* < .001), anal intercourse with condom (*r* = .40, *p* < .001), and anal intercourse without condom (*r* = .43, *p* < .001). MWOH demonstrated similar correlations as average use of marijuana and ED drugs were weakly correlated (*r* = .19, *p* = .005), and stimulant use with ED drugs showed a slightly higher but weak correlation (*r* = .22, *p* = .002). Multiple partners had a moderate relationship with ED drug use over time (*r* = .36, *p* < .001), and anal intercourse with condom (*r* = .22, *p* = .002) and anal intercourse without condom (*r* = .18, *p* = .01) also demonstrated positive correlations.

**Table 4:** Generalized linear mixed models with ED drug use and a second outcome variable using bivariate random-intercept model in MWH and MWOH

Outcome variable	MWH	Outcome variable	MWOH
	*r*		*r*
Marijuana	.19***	Marijuana	.19**
Poppers	.27***		
Stimulants	.25***	Stimulants	.22**
Testosterone	.32***		
Depression medication	.08*	Diabetes medication	− .06
ART	.03		
Multiple partners	.41***	Multiple partners	.36***
Insertive anal intercourse with condom	.40***	Insertive anal intercourse with condom	.22**
Insertive anal intercourse without condom	.43***	Insertive anal intercourse without condom	.18*

Models were adjusted for age, race, education, smoking status, alcohol consumption, BMI, kidney disease, HCV infection, pre-existing conditions

Models that did not converge are not presented in the table. Standard errors (confidence intervals) are not given since it could not be computed from the software

*MWH* men with HIV, *MWOH* men without HIV, *ART* antiretroviral therapy, *r* correlation coefficient

*p < .05. **p < .01. ***p < .001

Among MWH, use of marijuana (*b* = .19, *p* < .001), poppers (*b* = .28, *p* < .001), stimulants (*b* = .40, *p* < .001), and testosterone (*b* = .40, *p* < .001) exhibited statistically significant associations with visit-level ED drug use (Table 5). Multiple partners (*b* = .59, *p* < .001), anal intercourse with condom (*b* = .72, *p* < .001), and anal intercourse without condom (*b* = .64, *p* < .001) also increased with increasing ED drug use over time. In the MWOH group, using recreational drugs was not associated with ED usage at the visit-level within individuals, only the sexual behavior variables; i.e., multiple partners (*b* = .55, *p* < .001), anal intercourse with condom (*b* = .64, *p* = .002) and anal intercourse without condom (*b* = .51, *p* = .01) were associated with ED drug use.

**Table 5:** Generalized linear mixed model using ED drug residuals and outcome variable in a bivariate random-intercept model for associations at an individual level over time

Outcome variable	MWH		MWOH	
	Estimate	SE	Estimate	SE
Marijuana	.19***	.05	.16	.12
Poppers	.28***	.05		
Stimulants	.40***	.06	.13	.17
Testosterone	.40***	.06		
Depression medication	.17***	.05		
Diabetes medication			.15	.18
ART	− .02	.06		
Multiple partners	.59***	.05	.55***	.11
Insertive anal intercourse with condom	.72***	.05	.64***	.12
Insertive anal intercourse without condom	.64***	.05	.51***	.13

Residual = (Observed proportion of ED drug use – Expected proportion of ED drug use). Computed from generalized linear mixed models adjusted for age, race, education, smoking status, alcohol consumption, BMI, HIV status, kidney disease, HCV infection, pre-existing conditions

Models that did not converge are not presented in the table

*MWH* men with HIV, *MWOH* men without HIV, *ART* antiretroviral therapy, *SE* standard error

*p < .05. **p < .01. ***p < .001

## Discussion

In this large and longitudinally well-characterized group of MWH and MWOH MSM, we found that use of marijuana, poppers, stimulants, and testosterone was correlated with ED drug use. Likewise, depression medication usage was weakly positively correlated with ED drug use. Furthermore, ED drug use and engagement in sexual activities, such as having more than two partners since last visit, as well as reporting insertive anal intercourse with and without condom, increased together over time.

Our findings were consistent with those of other studies that examined associations between ED drug usage, recreational drug use, and lifetime sexual partners (Chan et al., 2015; Fisher et al., 2006; Garin et al., 2017; Halkitis & Green, 2007; Harte & Meston, 2012; Paul et al., 2005). Our findings regarding the association between ED drug use and insertive anal intercourse without condom corroborated those from a study by Schwarcz et al. (2007) and Mitchell et al. (2016). Fisher et al. (2011) noted that men who were using recreational drugs were more likely to be using ED drugs; they also reported an association between ED drug use and GHB and cocaine use among men who were taking both sildenafil and methamphetamine, while another study found that users of sildenafil, along with other drugs, reported higher numbers of sex partners than those who did not use them together (Kim et al., 2002). In a descriptive analysis of users of both marijuana and ED drugs, men used ED drugs to offset the impact of marijuana on libido, since marijuana acts on the same cytochrome P450 enzyme as sildenafil (Eloi-Stiven et al., 2007; Hubbard et al., 1999). Furthermore, men were more likely to be engaged in anal intercourse without condom if they were using more than two drugs simultaneously (Halkitis & Parsons, 2002), and sildenafil users were more likely to have condomless anal sex (Swearingen & Klausner, 2005).

Within individuals, on average, use of ED drugs and other recreational drugs was positively associated. Use of testosterone, depression medication, and sexual behaviors were associated with visit-level within-person ED drug use. This suggests that increased ED drug use on average was associated with increased use of recreational drugs and sexual behaviors within individuals. To our knowledge, there are no studies to date that have examined patterns of ED drug use in association with other behavioral factors at an individual level. One study that investigated the longitudinal pattern of recreational drug use and sexual behavior among MSM found that even sporadic use of drugs was associated with a higher risk of engaging in unprotected anal sex (Colfax et al., 2005). Although the study did not look at ED drugs, it did suggest that some of the recreational drugs used alone contributed to sexual behaviors, thereby increasing the risk of HIV transmission. Most studies that have assessed the use of ED drugs, recreational drugs, and sexual behaviors have suggested that an intervention on one of the factors may not be enough to help reduce transmission. Our study supports such a hypothesis, since concurrent use of ED and recreational drugs, as well as sexual behavior, was positively associated over time.

The strengths of our study include the use of a large, well-defined longitudinal cohort. Most prior studies were of cross-sectional design, or only evaluated baseline characteristics without examining ED drug use longitudinally. In the MACS, ED drug use was assessed longitudinally, and included socially sensitive data collected through a standardized method for data collection. The statistical method used in our analysis has several advantages. The bivariate random-intercept model shows whether average person-level usage of two variables are correlated, as well as whether these variables move up and down together at the visit-level within the individual. Joint modeling of the two variables as a bivariate response that was repeatedly measured over time allowed for such analysis. In addition, variables with different numbers of observations or different times of observation can be analyzed by this model.

Our findings have several limitations. We could not evaluate ED drug frequency or dosage information. Likewise, we could not separately evaluate specific ED drug usage, i.e., we could not determine whether ED drug use was for prescription or recreational purposes. Prescription drug information was not available, so ED drug use by participants could not be linked to confirm self-reported responses. Hence, we could not perform subgroup analysis for prescription and recreational use of ED drugs. Social demographic information, as well as data regarding recreational drug use and sexual practices, were also from self-reported data. Even though the use of audio computer-assisted self-interviewing (ACASI) helps participants respond to sensitive questions more accurately, there can still be misreporting of information. Finally, sexual partner HIV serostatus was usually unknown, data on sexually transmitted infections were not collected, and pre-exposure prophylaxis (PrEP), which was approved by the FDA in 2012, was unavailable for most of our study period. Therefore, we could not determine whether insertive anal intercourse with or without condom was in the context of PrEP use.

### Public Health Implications

Our study showed a positive relationship between ED drug use with other outcomes, including use of marijuana, poppers and engaging in insertive anal intercourse without condom. Furthermore, our findings suggest a positive association between ED drug use over time and use of other recreational drugs. Sexual behaviors, such as having multiple partners, also increased with average ED drug use.

It is unsafe to administer ED drugs with nitrates/nitrites and with protease inhibitor antiretroviral medications because: (1) concurrent use of sildenafil and nitrite inhalers (poppers) could lead to hypotension; (2) concurrent use of protease inhibitors generally increases the bioavailability of concurrently used ED drugs, necessitating downward dosing of the latter (James, 1998; Lim et al., 2002); (3) many MWH used poppers and/or were on antiretroviral therapy while using ED drugs. Therefore, monitoring the use of all of these drugs may be useful to inform the planning and promotion of interventions for healthy behaviors. Men taking ED drugs need to be informed about drug interactions and sex behaviors (Chu et al., 2003; Kim et al., 2002). In at least two reports, ED drug usage was associated with the risk of seroconversion (Ostrow et al., 2009; Prestage et al., 2009), and its use in combination with recreational drugs and sexual behaviors may have even greater detrimental effects, particularly among MSM. As has been suggested in prior studies (Baker et al., 2019; Chu et al., 2003; Garin et al., 2017; Ryan et al., 2018; Swearingen & Klausner, 2005), we may need to provide better public health guidance to persons who use ED drugs, keeping in mind that some persons obtain ED drugs from non-prescriber sources for recreational use. With aging, the use of ED drugs will increase among sexually active men, and intervention strategies may be needed to prevent HIV transmission.

### Conclusion

The use of ED drugs was positively correlated with use of other recreational drugs, both at the population level and at the visit level within individuals over time. Furthermore, increases in average ED drug use over time was associated with increases in sexual behavior, such as having multiple partners and insertive anal intercourse with and without condom, among MSM, regardless of HIV serostatus.

## References

[CR1] Baker JC, Fintelmann R, Sharifi R, Lee M (2019). Precautions and monitoring of patients taking phosphodiesterase type 5 inhibitors who are at risk of increased intraocular pressure. Drugs and Aging.

[CR2] Bechara A, Casabe A, De Bonis W, Helien A, Bertolino MV (2010). Recreational use of phosphodiesterase type 5 inhibitors by healthy young men. Journal of Sexual Medicine.

[CR3] Center for Disease Control and Prevention. (2017). *About adult BMI*. Retrieved from https://www.cdc.gov/healthyweight/assessing/bmi/adult_bmi/index.html

[CR4] Chan WL, Wood DM, Dargan PI (2015). Significant misuse of sildenafil in London nightclubs. Substance Use & Misuse.

[CR5] Chu PL, McFarland W, Gibson S, Weide D, Henne J, Miller P, Schwarcz S (2003). Viagra use in a community-recruited sample of men who have sex with men, San Francisco. Journal of Acquired Immune Deficiency Syndromes.

[CR6] Colfax G, Coates TJ, Husnik MJ, Huang Y, Buchbinder S, Koblin B, Team ES (2005). Longitudinal patterns of methamphetamine, popper (amyl nitrite), and cocaine use and high-risk sexual behavior among a cohort of san francisco men who have sex with men. Journal of Urban Health.

[CR7] Detels R, Phair JP, Saah AJ, Rinaldo CR, Murioz A, Kaslow RA, Vermund S (1992). Recent scientific contributions to understanding HIV/AIDS from the multicenter AIDS cohort study. Journal of Epidemiology.

[CR8] Dijkstra M, van Lunsen RHW, Kooij KW, Davidovich U, van Zoest RA, Wit F, Group AGCS (2018). HIV-1 status is independently associated with decreased erectile function among middle-aged MSM in the era of combination antiretroviral therapy. AIDS.

[CR9] Eloi-Stiven ML, Channaveeraiah N, Christos PJ, Finkel M, Reddy R (2007). Does marijuana use play a role in the recreational use of sildenafil?. Journal of Family Practice.

[CR10] Ferri C, Bertozzi MA, Zignego AL (2002). Erectile dysfunction and hepatitis C virus infection. Journal of the American Medical Association.

[CR11] Fisher DG, Malow R, Rosenberg R, Reynolds GL, Farrell N, Jaffe A (2006). Recreational viagra use and sexual risk among drug abusing men. American Journal of Infectious Diseases.

[CR12] Fisher DG, Reynolds GL, Ware MR, Napper LE (2011). Methamphetamine and viagra use: Relationship to sexual risk behaviors. Archives of Sexual Behavior.

[CR13] Garin N, Zurita B, Velasco C, Feliu A, Gutierrez M, Masip M, Mangues MA (2017). Prevalence and clinical impact of recreational drug consumption in people living with HIV on treatment: A cross-sectional study. British Medical Journal Open.

[CR14] Halkitis PN, Green KA (2007). Sildenafil (Viagra) and club drug use in gay and bisexual men: The role of drug combinations and context. American Journal of Men's Health.

[CR15] Halkitis PN, Green KA, Mourgues P (2005). Longitudinal investigation of methamphetamine use among gay and bisexual men in New York City: Findings from Project BUMPS. Journal of Urban Health.

[CR16] Halkitis PN, Parsons JT (2002). Recreational drug use and HIV-risk sexual behavior among men frequenting gay social venues. Journal of Gay & Lesbian Social Services: Issues in Practice, Policy & Research.

[CR17] Harte CB, Meston CM (2011). Recreational use of erectile dysfunction medications in undergraduate men in the United States: Characteristics and associated risk factors. Archives of Sexual Behavior.

[CR18] Harte CB, Meston CM (2012). Recreational use of erectile dysfunction medications and its adverse effects on erectile function in young healthy men: The mediating role of confidence in erectile ability. Journal of Sexual Medicine.

[CR19] Hubbard JR, Franco SE, Onaivi ES (1999). Marijuana: medical implications. American Family Physician.

[CR20] James, J. S. (1998). Viagra warning re "poppers" and notice re protease inhibitors. *AIDS Treat News *(no. 294), 1. Retrieved from https://www.ncbi.nlm.nih.gov/pubmed/11365402 11365402

[CR21] Kaslow RA, Ostrow DG, Detels R, Phair JP, Polk BF, Rinaldo CR (1987). The multicenter AIDS cohort study: Rationale, organization, and selected characteristics of the participants. American Journal of Epidemiology.

[CR22] Kim AA, Kent CK, Klausner JD (2002). Increased risk of HIV and sexually transmitted disease transmission among gay or bisexual men who use Viagra, San Francisco 2000–2001. AIDS.

[CR23] Lim PH, Moorthy P, Benton KG (2002). The clinical safety of viagra. Annals of the New York Academy of Sciences.

[CR24] Mansergh G, Shouse RL, Marks G, Guzman R, Rader M, Buchbinder S, Colfax GN (2006). Methamphetamine and sildenafil (Viagra) use are linked to unprotected receptive and insertive anal sex, respectively, in a sample of men who have sex with men. Sexually Transmitted Infections.

[CR25] Miranda-Sousa AJ, Davila HH, Lockhart JL, Ordorica RC, Carrion RE (2006). Sexual function after surgery for prostate or bladder cancer. Cancer Control.

[CR26] Mitchell KR, Prah P, Mercer CH, Datta J, Tanton C, Macdowall W, Wellings K (2016). Medicated sex in Britain: Evidence from the third national survey of sexual attitudes and lifestyles. Sexually Transmitted Infections.

[CR27] Ostrow DG, Plankey MW, Cox C, Li X, Shoptaw S, Jacobson LP, Stall RC (2009). Specific sex drug combinations contribute to the majority of recent HIV seroconversions among MSM in the MACS. Journal of Acquired Immune Deficiency Syndromes.

[CR28] Paul JP, Pollack L, Osmond D, Catania JA (2005). Viagra (sildenafil) use in a population-based sample of US men who have sex with men. Sexually Transmitted Diseases.

[CR29] Prestage G, Jin F, Kippax S, Zablotska I, Imrie J, Grulich A (2009). Use of illicit drugs and erectile dysfunction medications and subsequent HIV infection among gay men in Sydney, Australia. Journal of Sexual Medicine.

[CR30] Purcell DW, Moss S, Remien RH, Woods WJ, Parsons JT (2005). Illicit substance use, sexual risk, and HIV-positive gay and bisexual men: Differences by serostatus of casual partners. AIDS.

[CR31] Purcell DW, Wolitski RJ, Hoff CC, Parsons JT, Woods WJ, Halkitis PN (2005). Predictors of the use of viagra, testosterone, and antidepressants among HIV-seropositive gay and bisexual men. AIDS.

[CR32] Romero-Velez G, Lisker-Cervantes A, Villeda-Sandoval CI, Sotomayor de Zavaleta M, Olvera-Posada D, Sierra-Madero JG, Castillejos-Molina RA (2014). Erectile dysfunction among HIV patients undergoing highly active antiretroviral therapy: Dyslipidemia as a main risk factor. Sexual Medicine.

[CR33] Ruf M, Lovitt C, Imrie J (2006). Recreational drug use and sexual risk practice among men who have sex with men in the United Kingdom. Sexually Transmitted Infections.

[CR34] Ryan KE, Wilkinson AL, Pedrana A, Quinn B, Dietze P, Hellard M, Stoove M (2018). Implications of survey labels and categorisations for understanding drug use in the context of sex among gay and bisexual men in Melbourne, Australia. International Journal on Drug Policy.

[CR35] Schwarcz S, Scheer S, McFarland W, Katz M, Valleroy L, Chen S, Catania J (2007). Prevalence of HIV infection and predictors of high-transmission sexual risk behaviors among men who have sex with men. American Journal of Public Health.

[CR36] Spindler HH, Scheer S, Chen SY, Klausner JD, Katz MH, Valleroy LA, Schwarcz SK (2007). Viagra, methamphetamine, and HIV risk: Results from a probability sample of MSM, San Francisco. Sexually Transmitted Diseases.

[CR37] Swearingen SG, Klausner JD (2005). Sildenafil use, sexual risk behavior, and risk for sexually transmitted diseases, including HIV infection. American Journal of Medicine.

[CR38] Weiss R (2005). Modeling longitudinal data.

